# Use of a Thermodynamic Sensor in Monitoring Fermentation Processes in Gluten-Free Dough Proofing

**DOI:** 10.3390/s23010534

**Published:** 2023-01-03

**Authors:** Martin Adamek, Magdalena Zvonkova, Iva Buresova, Martin Buran, Veronika Sevcikova, Romana Sebestikova, Anna Adamkova, Nela Skowronkova, Jiri Mlcek

**Affiliations:** 1 Department of Automation and Control Engineering, Faculty of Applied Informatics, Tomas Bata University in Zlin, Nad Stranemi 4511, 760 05 Zlin, Czech Republic; 2 Department of Microelectronics, Faculty of Electrical Engineering and Communication, Brno University of Technology, Technicka 3058/10, 616 00 Brno, Czech Republic; 3 Department of Food Analysis and Chemistry, Faculty of Technology, Tomas Bata University in Zlin, Vavreckova 5669, 760 01 Zlin, Czech Republic; 4 Department of Food Technology, Faculty of Technology, Tomas Bata University in Zlin, Vavreckova 5669, 760 01 Zlin, Czech Republic

**Keywords:** thermodynamic sensor, optimization, rheofermentometer, e-nose, fermentation, gluten-free, dough, edible insects, the dough development, the production of gas

## Abstract

Dough fermentation in gluten-free bakery products is problematic due to the absence of gluten, which provides advantageous rheological properties. A thermodynamic sensor (TDS) system combined with an electronic nose was tested as an alternative to conventional methods monitoring dough development based on mechanical properties. In the first part, the configuration of the sensors in the thermodynamic system and their response to different heat-source positions, which significantly affect the output signal from the measurement system, were investigated. The practical contribution lies in the application of the measurements to the example of gluten-free doughs with and without edible insect enrichment. An optimized configuration of the thermodynamic system (one sensor on the inner wall of the container at the bottom and another in the middle of the container closer to the top of the dough) in combination with an experimental electronic nose was used for the aforementioned measurement. In some cases, up to 87% correlation between the signal from the TDS and the signals from a professional rheofermentometer Rheo F-4 (Chopin) was demonstrated. The differences between the results can be explained by the use of different techniques. Using a combination of sensor systems in one place, one time and one sample can lead to more comprehensive and robust results. Furthermore, it was shown that the fermentation activity increased in corn dough with the addition of insects compared to dough without the addition. In rice flour dough with the addition of edible insects, fermentation activity was similar to that of the flour without the addition.

## 1. Introduction

The idea of using thermodynamic sensors (TDS) in several industrial applications has been relevant for some time [1]. Among other research activities, this concept has been experimentally applied to the process of dairy production [2,3]. However, the application of these sensors has the potential to be used in a much wider range of food processes. In general, it can be argued that any process in which the heat flows out of the local thermodynamic system due to entropy is suitable for the application of this type of sensor system. One of the many examples are processes using fermentation [2,3,4]. A gluten-free dough fermentation suitable for people with celiac disease is used here as a model example.

Celiac disease is an autoimmune chronic disease affecting approximately 1% of the world’s population [5]. Although the diagnosis is now very efficient and much faster than in the past, the only compensation for celiac disease is still only the adherence to a gluten-free diet [5]. With the higher prevalence of diagnosed celiacs and also of people who avoid gluten by choice, this has opened up the market for the development of new products targeting these consumers. The preparation of gluten-free dough and consequently gluten-free bakery products presents a technological challenge, as gluten significantly affects the properties of bakery products [4,6]. Therefore, in the technology of gluten-free dough preparation, it is necessary to modify the input ingredients and the production process in order to achieve optimal properties of the final product. For this reason, many manufacturers use different types of alternative flours and additives in their recipes, such as hydrocolloids or alternative protein sources [6], which aim to mimic the viscoelastic properties of the gluten network, especially its ability to retain gas during leavening [7].

Compared to wheat dough, gluten-free doughs exhibit narrow regions of elastic, time-dependent viscoelastic and viscous deformations, higher values of elastic and viscous moduli, and high peak complex viscosity during heating, which have a significant deteriorating impact on the gluten-free dough’s ability to accumulate leavening gas in pores [8]. Gluten-free breads have, therefore, insufficient springiness, cohesiveness and resilience, low specific loaf volume, as well as short shelf life [9,10].

The popularity of insects in food applications is increasing not only due to the search for sustainable sources of wholesome proteins, but also because of the potential nutritional and health benefits that edible insects may present as a result of their high nutritional value [11]. Flour enrichment with edible insects is not only relevant for gluten-free doughs, but also for conventional wheat doughs for baking. The moderate addition of flour from different edible insects can improve the nutritional properties of the product, such as increasing the amino acid score (AAS) of lysine in conventional wheat doughs or enriching the essential fatty acids. The addition of edible insects also has an equally important effect on the textural and sensory properties of bakery products [12,13,14,15,16]. In the case of gluten-free flours, the addition of edible insects can achieve, as in the case of wheat doughs, not only nutritional enrichment but also acceptable technological properties [17], possibly improving some of the observed properties of the final product, such as consistency [18]. The fortification of doughs for the preparation of gluten-free baked goods with edible insects also affects the change of textural properties over time, since the insect meal slows down the hardening of the product during storage [19].

### 1.1. Scientific Hypotheses

**Hypothesis** **1.** *The location of the TDS sensors (thermodynamic sensors) will affect the output signal from the measurement system.*

**Hypothesis** **2.** *Monitoring the fermentation process with TDS (thermodynamic sensors) will correlate with the results from the rheofermentometer.*

**Hypothesis** **3.** *The addition of edible insects to gluten-free flour will affect the ability of the dough to produce and accumulate leavening gas.*

### 1.2. The Target

The aim of the work is to determine the influence of the arrangement of the TDS in the measuring vessel on the output response of the system, and to compare the course of fermentation of gluten-free doughs fortified with and without the addition of edible insects (0%; 5% and 10%) in an example configuration. The results obtained will be compared with similar measurements using a professional Rheo F4—Dough Proofing rheofermentometer, CHOPIN Technologies, Cedex, France.

## 2. Materials and Methods

### 2.1. Arrangement and Methodology of Measurements with TDS

**Figure 1:**
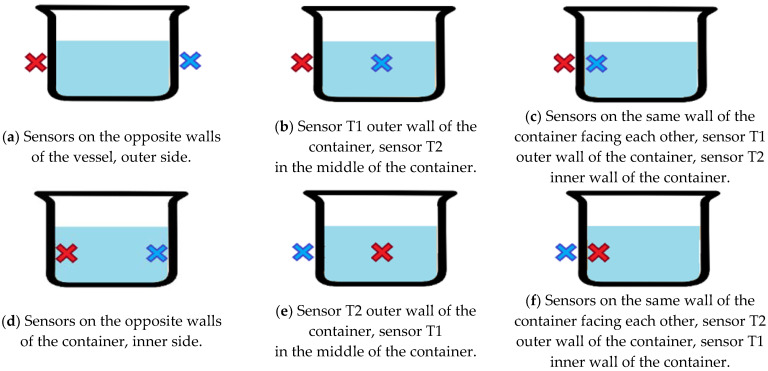
Schematic cases for different thermodynamic sensor configurations. The crosses show the position of the individual TDS sensors (the red cross indicates the T1 sensor, the blue cross indicates the T2 sensor) in relation to the measuring container with water.

The experiment was divided into three parts in accordance with each hypothesis. In the first part, the effect of the arrangement (position) of the sensors in the thermodynamic system on the output response of the TDS was investigated. The experimental setup for the measurements with thermodynamic sensors and the basic measurement procedure are described in [4,20]. A pilot experiment was performed with different positions of the thermodynamic sensors relative to the measuring vessel as shown in Figure 1.

Distilled water placed in a plastic container was used as the base material. To increase the internal temperature of the solution and the heat flow outwards, a certain smaller amount of water (0.5 L) was gradually added to the original batch of water in the vessel (6 L, approx. 20–23 °C) at the specific temperature (100 °C) at a casting speed of 0.025 L/s. As a result of the mentioned experiment, the solution was completely mixed within 1–2 min and the final temperature corresponded to the calorimetric Equation (1),

(1) $$c_{1}\cdot m_{1}\;\cdot \left(t_{1}-t\right)=c_{2}\cdot m_{2}\;\cdot \left(t-t_{2}\right),\;$$

where

*m*_1_ and *m*_2_ are the mass of substances,
*c*_1_ and *c*_2_ are specific heats of substances (liquid water 4.184 J⋅kg^−1^⋅K^−1^),
*t*_1_ and *t*_2_ are temperatures of substances before the thermal change,
*t* is final temperature of water in box after mixing.

In the first phase, the thermodynamic sensor system was configured so that the signal gain was as high as possible for the output voltage measuring the heat flow. This was achieved by comparing several possible configurations of thermodynamic sensors. Another essential and necessary step was to adapt the design of the sensors to the fermentation medium environment, for example, the minimum possible insulation in terms of heat-flow inertia which is also sufficient for measurement in the given environment.

**Figure 2:**
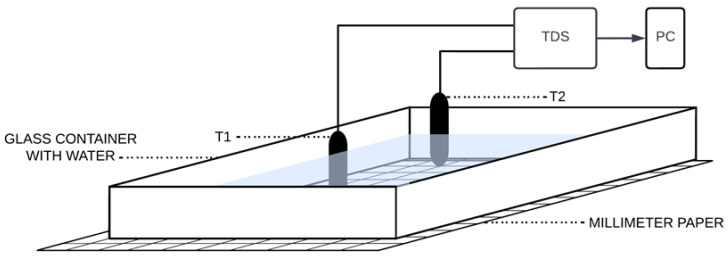
Schematic diagram of the arrangement of sensors in the thermodynamic system in the second part of the pilot experiment.

The second part of the pilot experiment was aimed at the determination of changes in output voltage depending on the position of the heat source at constant position of the TDS sensors. To determine the position, a coordinate system was chosen to be placed under the vessel (see Figure 2).

**Figure 3:**
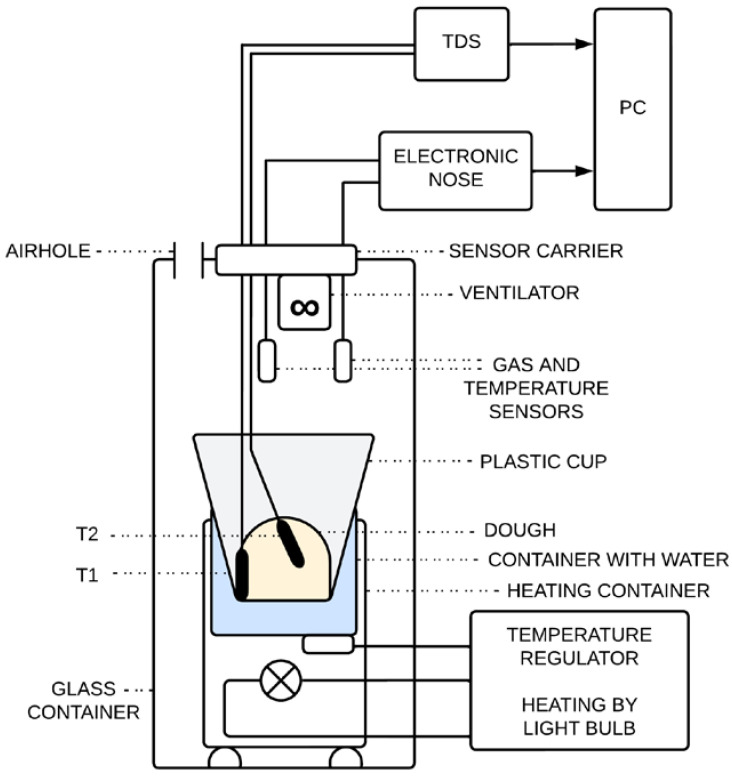
The schematic diagram of the fermentation measurement of various flour mixtures using thermodynamic sensors.

The monitoring of the fermentation process by the TDS was carried out in the configuration shown in Figure 3. A 0.2 L container with fermented dough is placed in a water bath whose temperature is controlled by a temperature controller. A 12V/5W bulb is used as a heating element, which is placed in a metal container adapted as a holder for the water bath. The whole system is housed in a larger glass container which separates it and protects it from the surrounding environment. A sensor carrier is attached to the neck of the glass container. On this carrier are placed not only the gas sensors of the electronic nose, but also the sensors of the thermodynamic system. These sensors are inserted into the fermenting dough. The output signals from the e-nose and the TDS are recorded by a computer, where they are then further processed.

### 2.2. Measuring on a Professional Rheofermentometer Rheo F4

To test the behavior of the dough during proofing and to compare the thermodynamic system with the standard laboratory method, a professional rheofermentometer Rheo F4—Dough Proofing, CHOPIN Technologies, Cedex, France was used. The instrument was set up as follows: Temperature 28.5 °C, Time 180 min, Dough weight 200 g, Constraint 0 g, mixer Eta Gratus (Eta, a.s. Czechia), Yeast quantity 12.5%. The standard Chopin protocol was used on the apparatus. Each test was performed on dough samples prepared in triplicate. The results are given as mean values.

### 2.3. Electronic Nose Measurement

The experimental setup for gas sensor (e-nose) measurements and the basic measurement procedure are described in [4,20]. The measurements were performed together with selected measurements using the TDS.

Because of the comparison of the nature (shape) of the individual output signals with each other, it was not necessary to determine the exact concentrations of the individual gases. Therefore, for simplicity, the output signals from the electronic nose sensors are given on a relative scale given by the output signal from the internal 10-bit A/D converter. In the case of the MQ-3, MQ-8 and MQ-135 sensors (Zhengzhou Winsen Electronics Technology Co., Ltd., Zhengzhou, China), the conversion is from voltage level U_out_ = 0–5 V to digital level d = 0–1023). In the case of the SGP30 sensor (Sensirion AG, Staefa ZH, Switzerland), the calculated TVOC signal is given in the range from 0 ppb to 60,000 ppb and the CO_2eq_ signal in the range from 400 ppm to 60,000 ppm. Both signals are calculated directly in the sensor from the raw signals for ethanol and H_2_.

### 2.4. Basic Ingredients

For the production of the dough, the recipe shown in Table 1 was chosen. The various basic ingredients are commonly available in supermarket chains.

### 2.5. Preparation of the Meal from Tenebrio molitor Larvae

The flour for the basic dough mixture was fortified with the addition of meal from the larvae of the mealworm (*Tenebrio molitor*) in the range of 0 and 10%. The larvae (full length of the body just before the pupae) were purchased from Radek Frýželka, Brno, Czech Republic. The larvae were separated into a separate box after purchase and starved for 48 h. They were then killed with boiling water (100 °C) and dried at 105 °C to constant weight. The obtained samples were homogenized for 1 mi by the coffee grinder Scarlett Silver Line SL-1545 (ARIMA, UK). The maximum size of the obtained particles was estimated to be below 1 mm. The samples were placed in a plastic bag and stored at 4–7 °C until use.

### 2.6. Recipe for the Preparation of the Basic Dough

TDS: For the preparation of the sourdough, the classic indirect dough management method was chosen, where the instant dry yeast is first activated before being added to the dough. Dry yeast was added to the heated milk (37 °C) and mixed with half of the sugar. The mixture was kept warm for about 15 min to allow the yeast to activate. Once the yeast was activated, the dry ingredients (flour, remaining sugar, salt) were mixed in an Eta Gratus mixer bowl (Eta, a.s. Czechia). The activated yeast and oil were added to these ingredients. The dough was mixed for 6 ± 1 min, 400 rpm, using a single bent dough hook. A fine, compact dough was kneaded with a kneader or cooker and then used for the measurements. In order not to limit the activity of the yeast, the amount of sugar and oil was adjusted from the original recipe [21]. The original recipe also contained 2 eggs, but these were not used in the experiment. The amount of milk was determined according to the results of preliminary experiments. The optimal water absorption of gluten-free flours has not been defined yet. The amount of milk was not increased with the increasing amount of edible insect flour to minimize the possible factors influencing the obtained results.

Rheofermentometer: The ingredients were placed into an Eta Exclusive Gratus mixer bowl (Eta, a.s. Czech Republic). The dough was mixed for 6 ± 1 min, 400 rpm, using a single bent dough hook. The prepared dough (500 g) was weighed and 200 g of the dough was hand-rounded until the dough formed a ball. Subsequently the dough was incubated for 180 min in a Rheofermentometer (Chopin, France) at a temperature of 28.5 °C. Gas release and dough development curves were recorded. The values of H’_m_: maximum height of the gas release curve; T_1_: time required to obtain H’_m_; T_x_: dough porosity time (time when the dough starts to loose CO_2_); Total volume: total volume of gas release in ml; Volume of CO_2_ lost: carbon dioxide volume in ml that the dough has lost during proofing (A_2_); Retention volume: carbon dioxide volume in ml still retained in the dough at the end of the test (A_1_); T_1_: maximum dough development time in hours and minutes; H_m_: maximum dough development height under stress; T_2_ and T’_2_: relative stabilization time at the maximum point located at a height of 0.88 Hm without being lower than Hm-6 mm; ΔT_2_ = T_2_ − T’_2_ = dough tolerance; h: dough development height at the end of the test; and (H_m_-h)/H_m_: % of drop in development after 3 h were evaluated.

For the TDS measurements, 50 g of dough was used; in the case of Rheo F4, 200 g of dough was used, in an adequate proportion to the original recipe. 

Experiments with fortified flour were prepared by replacing 5% and 10% of gluten-free flour with *Tenebrio molitor* meal. The summary of used mixtures with the addition of different amounts of edible insect meal and the description of samples is shown in Table 2.

### 2.7. Statistical Analysis

All data obtained from the measurements were processed and evaluated using Microsoft Excel 2019 (Microsoft Corporation, Redmond, WA, USA).

For the signals from the TDS, the start of the measurements (time and output voltage at the point when the dough was inserted into the system) was shifted to t_0_ = 0 s and U_out_ U_0_ = 8 V. Unless otherwise specified, each test was performed with at least three repetitions. To be consistent with the measurements from the professional reofermentometer, the measurement time was extended to 3 h.

The results of individual monitored quantities were expressed as the mean (M) ± standard deviation (SD). Significant differences between samples were determined by analysis of variance, considering differences significant at *p* < 0.05. This statistical analysis was performed with STATISTICA CZ version 12 (StatSoft, Inc., Tulsa, OK, USA). Shapiro–Wilk test of normality and Levene’s test of homogeneity were performed for all monitored samples. Where the comparison of the effect of fortification with the same flour in the dough base satisfied conditions for a *t*-test, a two-tailed *t*-test was performed (α = 0.05). Otherwise, the Mann–Whitney U test was used (α = 0.05). The curves (time series) for TDS and rheofermentometer were compared with each other using Pearson’s correlation coefficient.

## 3. Results and Discussion

### 3.1. Pilot Experiment

#### 3.1.1. Effect of Sensor Position on TDS Output Response

**Figure 4:**
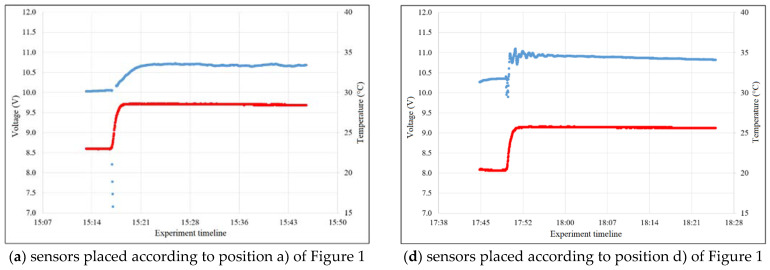

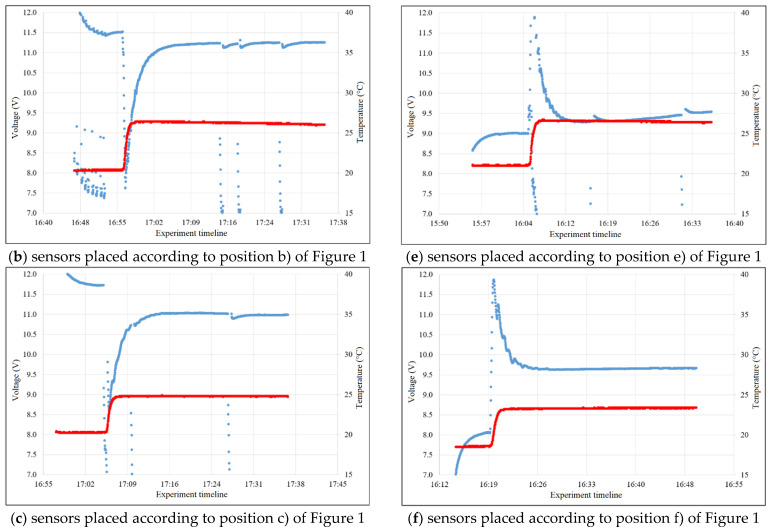
Thermal transfer characterization (blue curve—output signal from the TDS; red curve—water temperature) of sensor configurations shown in Figure 1.

The pilot experiment, which focused on the sensor arrangement of the thermodynamic system, showed a significant dependence of the maximum output signal on the location of these sensors in the thermodynamic system. Figure 4 shows the variation of the output signal according to the different configurations (positions of the sensors in the system) shown in Figure 1. The blue curve in Figure 1 shows the output signal from the TDS while the red curve represents water temperature which is stabilized at a certain value according to the calorimetric Equation (1).

Based on the previous experiment mentioned, configuration (e) was chosen as one of the best ones. Other configurations have proven to be less suitable or inappropriate (Figure 1a–d,f).

These results are only valid in the above circumstances, where a glass container with a specific sensor configuration is used. If the process takes place in a different material container, the heat flow properties between the sensors will be affected by the thermal properties of the container material. In other words, other sensor system configurations may be advantageous for different types of container materials. In some cases, the sensor system may be more sensitive;s in others, it may react with considerable delay or be prone to sensing ambient conditions.

#### 3.1.2. Effect of Signal Source Position on TDS Output Response

**Figure 5:**
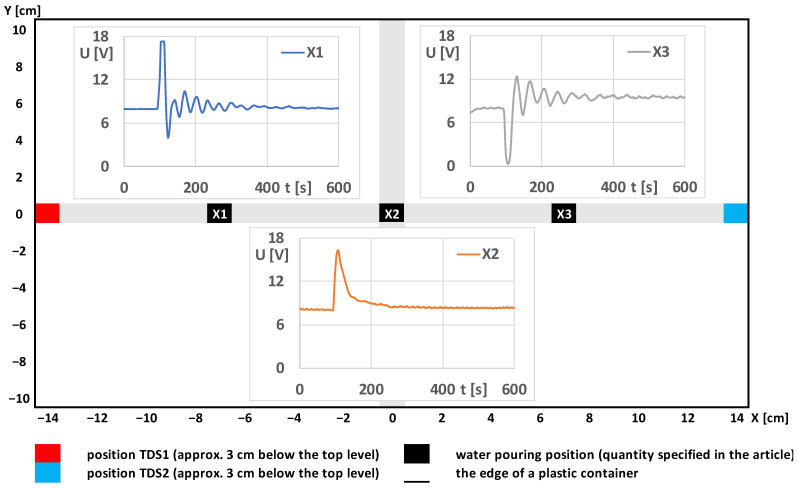
Changes in the output signal of the U_out_ thermodynamic system with fixed sensors at three different positions of hot water addition.

The next experiment was performed with the sensors of the thermodynamic system fixed on the edge of a plastic container. Hot water was added to the base temperature water bath vessel at various selected locations. The signal changes according to the three basic positions are shown in Figure 5.

**Figure 6:**
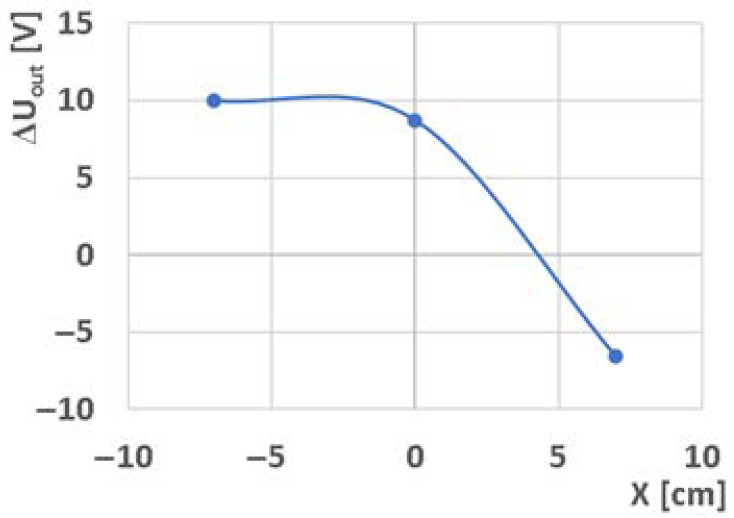
Dependence of the maximum change of the output signal ΔU_out_ (ΔU_out_ = U_outMAX_ − U_0_) of a thermodynamic system with fixed sensors on the change of hot water addition in the direct direction between the sensors.

After the basic signal processing (shifting the start of the measurement to the point t_0_ = 0 s and U_0_ = 8 V), the direction and maximum of the first signal waves were monitored. It is assumed that after the addition of hot water (temperature pulse) in the middle of the direct junction (position X2) between the technologically identically manufactured sensors, both sensors should be affected equally and the resulting change in output voltage should be 0 V. However, the orange curve for position X2 shows a change that demonstrates that the sensors do not have the same parameters, and the point at which there is no change in output voltage is shifted to sensor TDS2. This is illustrated by the graph in Figure 6, which shows the dependence of the magnitude of the peak on the position of the hot water addition. For the output signal at position X1, the maximum value of the U_out_ signal is limited by the measurement system. It is assumed that with a different system sensitivity and without this limitation, the curve in the graph in Figure 6 would be more linear.

Since few positions of the addition were measured and the limitation of the U_out_ signal was reached, the measured values should be taken as indicative. Nonetheless, they can be considered as initial data for further simulation and modelling of the TDS.

### 3.2. Dough-Rising Monitoring with Experimental TDS and Professional Rheo F4 Rheofermentometer

**Figure 7:**
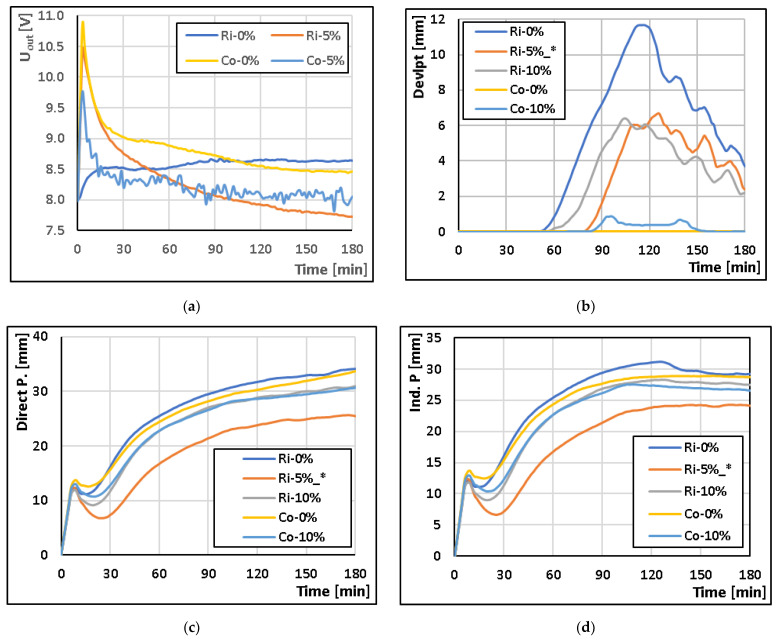
Comparison of the output signal U_out_ from the experimental TDS with the output signals from the professional Rheofermentometer RF-4 (Chopin, France): (**a**) Output signal U_out_ [V] from the TDS; (**b**) Dough development curve [mm]; (**c**) Gas release curve—Total volume; (**d**) Gas release curve—Retention volume.

The properties of the experimental thermodynamic measurement system were tested in the monitoring of gluten-free dough proofing. A basic type of flour (rice or corn) was fortified with meal from mealwort. The curves for the different flour types and fortification were measured 3 times. The exception is the Ri-5% type, where only 2 curves were processed on the rheofermentometer and must therefore be taken as indicative only (marked with *). The average values were then plotted on a graph. The results obtained are shown in Figure 7.

**Figure 8:**
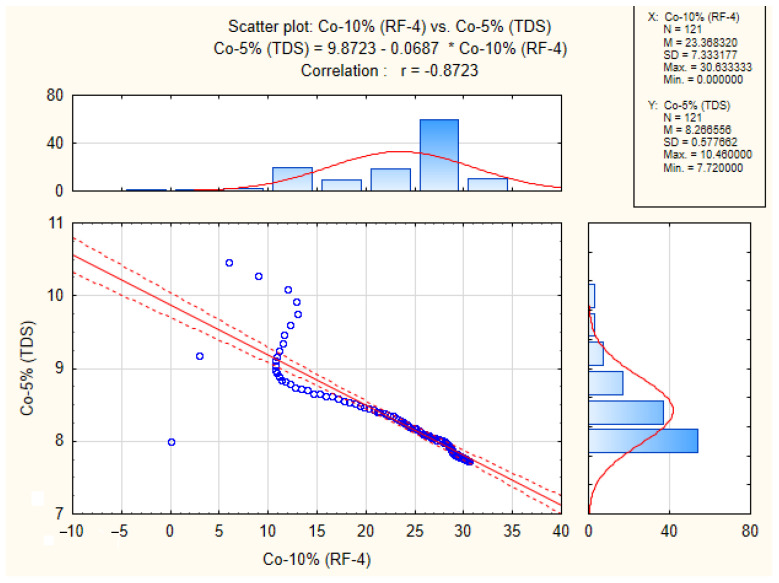
Example of correlation results from STATISTICA CZ version 12 (StatSoft, Inc., USA).

The correlation of the output signals between the different systems was determined using Pearson’s correlation coefficient, which is presented in Table 3 for the comparison of the different types of gluten-free dough. Since the Co-10% flour type was not measured on the TDS, this flour type measured on the RF-4 was compared to the Co-5% type measured on the TDS. All calculated coefficients are statistically significant at the significance level of *p* < 0.05. The table shows a low correlation between the output curves from the TDS and the dough development curve from RF-4, however, when compared to the gas release curve, the Pearson correlation coefficient rises to above 0.87. An example of the result from STATISTICA CZ version 12 (StatSoft, Inc., USA) is shown in Figure 8. It is clear from the result that if the initial values (initial wave) of the gas release curve are not taken into account, the correlation coefficient would be even higher.

### 3.3. Comparison of Dough with and without Edible Insect Fortification

The nature of the observed curves from the Rheo F4 professional rheofermentome-ter can be described by the significant bodies on this curve, as discussed in Section 2.6. Due to the increase in the height (volume) of the dough raised for corn flours after for-tification with edible insect meal, this thesis focused on this dough. The results from the dough development curve and gas production curve are shown in Table 4. In particular, the height of the developed gluten-free dough is very low. This may be because the standard method was designed to test the ability of wheat dough to produce and maintain leavening. The amount of water used to prepare gluten-free dough was higher than in wheat dough, which resulted in softer dough. The resistance weight (2 kg) used in the usual rheofermentometer analysis was removed as described by Gómez et al. (2013). The piston’s weight alone is, however, around 250 g. Because of this, the heights attained in the rheofermentometer are not easily comparable with the volume of the breads made using the same doughs, as this small weight has a greater effect on the less consistent doughs than on standardized wheat dough [22]. 

As it has been suggested by several authors, the addition of edible insect meal can influence bread volume and therefore improve the fermentation of dough. Kowalski et al. (2022) [12] studied the influence of buffalo worm, mealworm and cricket fortification. According to their results, buffalo worm flour (10% share) and mealworm flour (10 to 20% share) had a statistically significant effect on bread volume made from wheat flour. Similar results were reported by Roncolini et al. (2019) [16], who observed that a 5 to 10% addition of mealworm flour to wheat flour positively influenced bread volume. Vasilica et al. (2022) [23] described that edible insect meal can function not only as a nutritional enhancer for a wide range of food products but can also support the development and growth of certain bacterial strains, for example *Lactobacillus plantarum*. Naturally occurring lactic acid bacteria are an important part of the microbial ecosystem in bread sourdough [24], and enhancing their viability by the addition of edible insects could be also beneficial for the fermentation process itself.

The determinable values were then compared using a *t*-test. The results of the *t*-test for maize and rice flour are shown in Table 5. Although some values approached the value of 0.05, it was not reached in any of the tests. Therefore, the mean values of the measured traits are not statistically significantly different from each other. Similarly, the results for rice flour were similar. Here, the 0% and 10% groups were first compared using a *t*-test. Again, no statistically significant difference was found between the samples. Subsequently, the 5% group was added and the comparison was made using ANOVA. In this case, a statistically significant difference was observed for the group of H’m, Vt and Vr values. This comparison can only be taken as indicative as the measurements were only taken twice in this group and therefore it is not possible to perform the Shapiro–Wilk normality test. 

### 3.4. Comparison of Response from the TDS and Experimental e-Nose

In the last part of the work, the output response of U_out_ from the TDS was compared with the output response of the sensors from the experimental electronic nose on which the sensors from the thermodynamic system were placed. One sensor was placed touching the side of the container and the other one was placed as is described in Figure 1, configuration (e), which was considered the best.

**Figure 9:**
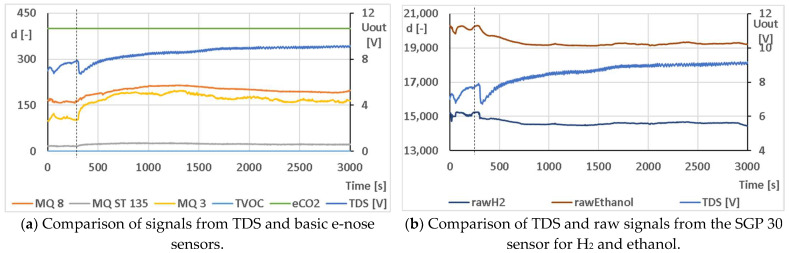
Example of comparison of output signals from the experimental e-nose and TDS for testing the budding of the addition of 0.5 g dried yeast (Thymos, spol. s r.o., Veľká Lomnica, Slovakia) in sugar solution (70 g/L). The moment when yeast was added into the sugar solution is marked by the dashed line.

A significant advantage of combining these systems was that the measurements could be made in a single instance and the individual signals could be accurately compared. The disadvantage was that the electronic nose uses a simple temperature controller, whose action is not detected by the gas sensors in the electronic nose but is responded to by the thermodynamic system. The whole system was tested on a sugar solution (70 g/L) with the addition of 0.5 g of dried yeast (Thymos, spol. s r.o., Veľká Lomnica, Slovakia). An example of the comparison of the output signals from the e-nose and TDS for this measurement is shown in Figure 9. The correlation coefficients for this example are given in Table 6. Since no change was detected in the TVOC and eCO_2_ signals throughout the measurement period, these signals could not be used for comparison and correlation coefficient calculation.

**Figure 10:**
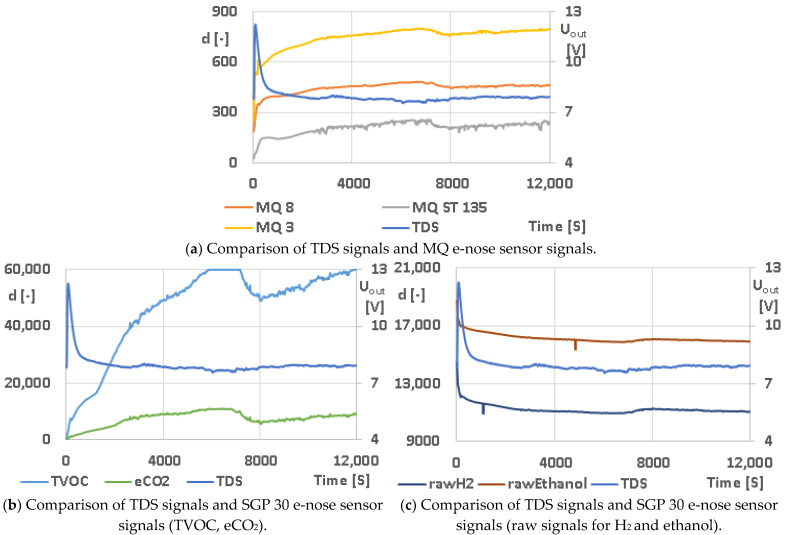
Co-0% gluten-free dough leavening—example of using the combined system of TDS and e-nose.

A Co-0% gluten-free dough leavening experiment was conducted in the suggested combined system of the TDS and e-nose. After the basic processing of acquired time series, the calculation of moving average (15 values, 30 s) was needed to removet the noise made by the operation of the temperature regulator. The examples of output values are shown in Figure 10 and correlation coefficients for the mentioned examples are listed in Table 7. Values show correlation between the signal from the TDS and the e-nose in the range between 60% and 73%.

## 4. Conclusions

This article deals with monitoring systems (mainly thermodynamic sensors) for gluten-free dough leavening fortified with insect meal (*Tenebrio molitor*) and optimises configurations of the thermodynamic sensor system. The article also compares the output values obtained from the TDS with those from a professional rheofermentograph and experimental e-nose.

It was proven that the sensor position in the suggested configurations significantly affected output signals of the thermodynamic sensor system. However, even in a constant configuration the influence of heat-source position was noted. The configuration where one sensor is placed inside in the center of the container and the other one is placed by the side was evaluated as the best one. While the correlation within these systems seems to be low, the advantage lies in obtaining various characteristics regarding leavening dough.

Final characteristics from the TDS were compared to values obtained from the professional rheofermentograph RF-4 (Chopin). In some cases, the results correlated with each other up to 87%. The differences between the results can be explained due to the use of different techniques.

Furthermore, it was demonstrated that the addition of edible insect meal into the dough made from corn flour enhanced the maximum development reached by the dough, correlated with bread volume. In contrast, an opposite effect of the addition of *T. molitor* meal to the dough made from rice flour was observed; thus in this case, the addition is not desirable with respect to leavening of the dough. On the other hand, nutritional value can be increased by edible insect fortification. 

Our research brings possible expansion of e-nose techniques by adding other measuring systems (in this case, a TDS). Using a combination of sensor systems in one place, one time and one sample can lead to more comprehensive and robust results. 

## Figures and Tables

**Table 1:** Content of ingredients used to monitor the fermentation of a basic dough.

Amount	Ingredients	Producer
500 g	Universal gluten-free baking mix (Nature’s Promise Bio) *	HAMMERMÜHLE, KIRRWEILER, Německo
	Plain rice flour *	Extrudo Bečice s.r.o., Bečice, Czech Republic
	Plain corn flour (Nature’s Promise Bio) *	PRO-BIO, Obchodní společnost S.R.O., Staré Město, Czech Republic
25 mL	Sunflower oil	Bunge Zrt., Budapest, Hungary
375 mL	Long-life whole milk	Mlékárna Pragolaktos, a.s., Prague, Czech Republic
100 g	Granulated sugar	Tereos TTD, Dobrovice, Czech Republic
25 g	Saf-instant instant yeast (1 × 500 g packet; gluten-free, lactose-free and sugar-free)	S.I.L. 137 rue Gabriel Péri, 59703 Marcq-en-Baroeul
3 g	Solsanka^®^ Sea salt with iodine and fluorine	Solsan, a.s., Prague, Czech Republic
0–10%	Edible insect flour (see Table 2)	

* Only one type of flour was used according to the type of dough required.

**Table 2:** Designation of individual mixtures, type and quantity of individual flours used for expe-riments.

Designation	Basic Flour Type	Insect Meal Content
Co-0%	Corn	0%
Co-5%	Corn	5%
Co-10%	Corn	10%
Ri-0%	Rice	0%
Ri-5%	Rice	5%
Ri-10%	Rice	10%

**Table 3:** Correlation of curves (time series) measured with the experimental TDS and professional Rheofermentometer RF-4.

1. Curve	2. Curve	Pearson Correlation Coefficient
Dough development curve		
Ri-0% (RF-4)	Ri-0% (TDS)	−0.618404265
Ri-10% (RF-4)	Ri-5% (TDS)	−0.549489715
Co-0% (RF-4)	Co-0% (TDS)	CNBD *
Co-10% (RF-4)	Co-5% (TDS)	−0.418418132
Gas release curve—Total volume		
Ri-0% (RF-4)	Ri-0% (TDS)	−0.787737988
Ri-10% (RF-4)	Ri-5% (TDS)	−0.718690282
Co-0% (RF-4)	Co-0% (TDS)	0.866863649
Co-10% (RF-4)	Co-5% (TDS)	−0.872280125
Gas release curve—Retention volume		
Ri-0% (RF-4)	Ri-0% (TDS)	−0.760860732
Ri-10% (RF-4)	Ri-5% (TDS)	−0.712353433
Co-0% (RF-4)	Co-0% (TDS)	0.878552143
Co-10% (RF-4)	Co-5% (TDS)	−0.853351929

* Cannot be determined.

**Table 4:** Results (M ± SE) from the dough development curve and gas production curve for corn flour without (Co-0%) and with the addition of 10% of edible insects (Co-10%) using the device rheofermentometer RF-4 (Chopin, France).

Parameters	Co-0%	Co-10%
Dough development curve		
Hm [mm]	0.0 ± 0.0	1.4 ± 0.7
h [mm]	0.0 ± 0.0	0.0 ± 0.0
(Hm-h)/Hm [%]	23 ± 21	67 ± 33
T1 [min]	178 ± 1	138 ± 25
T2 [min]	CNBD *	CNBD *
T’2 [min]	CNBD *	CNBD *
T2-T’2 [min]	CNBD *	CNBD *
Gas release curve		
H’m [mm]	33.7 ± 0.8	30.6 ± 1.9
T’1 [min]	180.0 ± 0.0	180.0 ± 0.0
Tx [min]	CNBD *	CNBD *
Vt [mL] (Total)	715 ± 22	659 ± 47
Vr [mL] (Retention)	681 ± 20	631 ± 42
Vc [mL] (CO_2_)	34 ± 3	29 ± 6
Vr/Vt [%] (CR)	95 ± 1	96 ± 1

* Cannot be determined.

**Table 5:** Results of *t*-test for corn and rice flour without (0%) and with the addition of 10% of edible insects flour using the rheofermentometer Rheo F-4 device (Chopin, France).

Parameters	Corn Flour				Rice Flour			
	M_Co-0%_	M_Co-10%_	t	*p*	M_Ri-0%_	M_Ri-10%_	t	*p*
Dough development curve								
Hm [mm]	0.0	1.4	−2.152	0.098	12.23	6.8	1.228	0.287
h [mm]	0.0	0.03	−1.000	0.374	3.7	2.2	0.701	0.522
(Hm-h)/Hm [%]	23.2	66.7	−1.099	0.333	70.87	74.93	−0.244	0.819
T1 [min]	178.0	137.5	1.610	0.183	110.5	103	0.414	0.700
Gas release curve								
H’m [mm]	33.7	30.6	1.512	0.205	34.2	31.0	1.674	0.169
Vt [mL] (Total)	715.3	659.3	1.074	0.343	737.3	658.7	1.746	0.156
Vr [mL] (Retention)	680.7	631.3	1.060	0.349	706.3	637.3	1.887	0.132
Vc [mL] (CO_2_)	34.3	28.7	0.909	0.415	31	21.3	1.028	0.362
Vr/Vt [%] (CR)	95.2	95.7	−0.930	0.405	95.9	96.8	−0.862	0.437

**Table 6:** Correlation coefficients for the comparison of output signals from the experimental e-nose and TDS testing for the budding of the addition of 0.5 g of dried yeast (Thymos, spol. s r.o., Veľká Lomnica, Slovakia) in sugar solution (70 g/L) from Figure 9. Calculated in STATISTICA CZ version 12 (StatSoft, Inc., USA).

Variable	Correlation Labeled Correlations Are Significant at the *p* < 0.05000 Level N = 2708 (Full Cases Omitted for ChD)							
	M	SD	TDS [V]	MQ 8	MQ ST 135	MQ 3	Raw H_2_	Raw Ethanol
TDS [V]	8.86	0.61	1.00	0.52	0.13	0.27	−0.80	−0.70
MQ 8	198.66	11.98	0.52	1.00	0.82	0.89	−0.84	−0.91
MQ ST 135	22.84	2.14	0.13	0.82	1.00	0.92	−0.54	−0.73
MQ 3	165.83	18.40	0.27	0.89	0.92	1.00	−0.64	−0.85
raw H_2_	14,561.11	182.19	−0.80	−0.84	−0.54	−0.64	1.00	0.89
raw ethanol	19,325.57	245.37	−0.70	−0.91	−0.73	−0.85	0.89	1.00

**Table 7:** Correlation coefficient—s for Co-0% gluten-free dough leavening corresponding to Figure 10. Calculated in STATISTICA CZ version 12 (StatSoft, Inc., USA).

Variable	Correlation Labeled Correlations Are Significant at the *p* < 0.05000 Level N = 7376 (Entire Cases Omitted for ChD)									
	M	SD	TDS [V]	MQ 8	MQ ST 135	MQ 3	TVOC	eCO_2_	Raw H_2_	Raw Ethanol
TDS [V]	7.96	0.47	1.00	−0.73	−0.65	−0.66	−0.60	−0.61	0.69	0.66
MQ 8	451.12	31.73	−0.73	1.00	0.94	0.97	0.91	0.89	−0.97	−0.97
MQ ST 135	215.39	35.63	−0.65	0.94	1.00	0.94	0.95	0.91	−0.89	−0.94
MQ 3	760.42	58.04	−0.66	0.97	0.94	1.00	0.95	0.87	−0.95	−0.99
TVOC	49,028.01	14,770.10	−0.60	0.91	0.95	0.95	1.00	0.91	−0.85	−0.94
eCO_2_	7824.22	2359.49	−0.61	0.89	0.91	0.87	0.91	1.00	−0.86	−0.88
raw H_2_	11,179.76	297.66	0.69	−0.97	−0.89	−0.95	−0.85	−0.86	1.00	0.95
raw ethanol	16,087.07	294.93	0.66	−0.97	−0.94	−0.99	−0.94	−0.88	0.95	1.00

## Data Availability

New research data were presented in this contribution.
